# Recycling pathways in cold-water coral reefs: Use of dissolved organic matter and bacteria by key suspension feeding taxa

**DOI:** 10.1038/s41598-020-66463-2

**Published:** 2020-06-18

**Authors:** Sandra R. Maier, Tina Kutti, Raymond J. Bannister, James Kar-Hei Fang, Peter van Breugel, Pieter van Rijswijk, Dick van Oevelen

**Affiliations:** 1 Department of Estuarine and Delta Systems, Royal Netherlands Institute for Sea Research (NIOZ-Yerseke) and Utrecht University, Yerseke, The Netherlands; 2 IMR Institute of Marine Research, Nordnesgaten 50, 5005 Bergen, Norway; 3 Department of Applied Biology and Chemical Technology, The Hong Kong Polytechnic University, Hung Hom, Kowloon Hong Kong

**Keywords:** Coral reefs, Carbon cycle, Stable isotope analysis, Marine biology

## Abstract

Cold-water coral (CWC) reefs are one of the most diverse and productive ecosystems in the deep sea. Especially in periods of seasonally-reduced phytodetritus food supply, their high productivity may depend on the recycling of resources produced on the reef, such as dissolved organic matter (DOM) and bacteria. Here, we demonstrate that abundant suspension feeders *Geodia barretti* (high-microbial-abundance sponge), *Mycale lingua* (low-microbial-abundance sponge) and *Acesta excavata* (bivalve) are able to utilize ^13^C-enriched (diatom-derived) DOM and bacteria for tissue growth and respiration. While DOM was an important potential resource for all taxa, utilization of bacteria was higher for the sponges as compared to the bivalve, indicating a particle-size differentiation among the investigated suspension feeders. Interestingly, all taxa released ^13^C-enriched particulate organic carbon, which in turn may feed the detritus pathway on the reef. Especially *A. excavata* produced abundant (pseudo-)fecal droppings. A second stable-isotope tracer experiment revealed that detritivorous ophiuroids utilized these droppings. The high resource flexibility of dominant reef suspension feeders, and the efficient recycling of their waste products by the detritivore community, may provide important pathways to maintain the high productivity on cold-water coral reefs, especially in periods of low external food supply.

## Introduction

**Figure 1:**
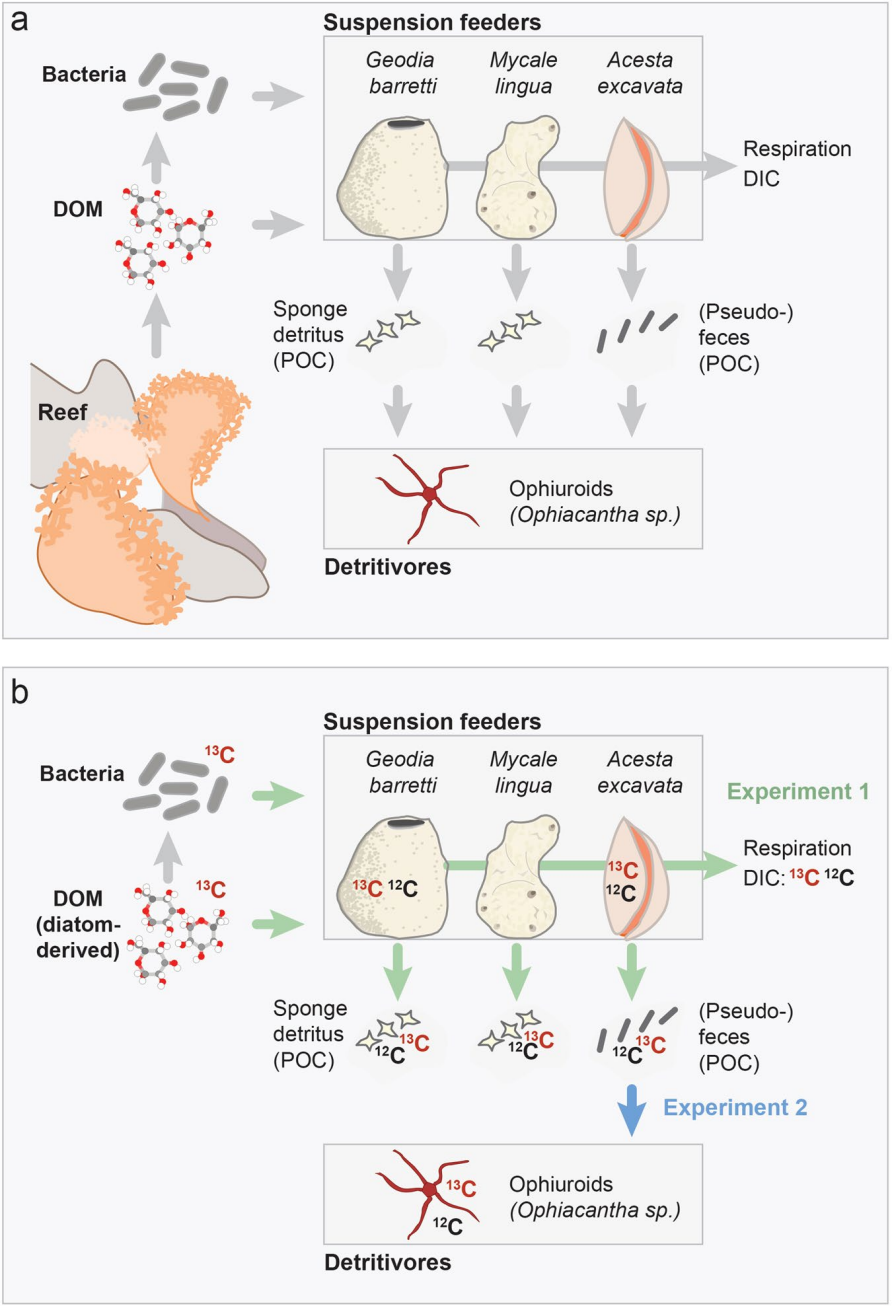
Potential recycling pathways on cold-water coral reefs, and experimental investigation. (**a**) Suspension feeders on the reef may exploit enhanced concentrations of dissolved organic matter (DOM) and bacteria, transfer it to tissue biomass, and utilize it for respiration and the production of particulate waste such as sponge detritus or bivalve (pseudo-)feces. Particulate waste material may be recycled by reef detritivores. (**b**) The indicated hypothesized ‘recycling’ pathways (green and blue arrows) were verified in two laboratory stable isotope ^13^C-tracer experiments, using the artificially ^13^C-enriched substrates DOM (diatom-derived), bacteria, and *A. excavata* (pseudo-)feces.

Cold-water coral (CWC) reefs^1^ rank amongst the most metabolically-active ecosystems of the deep sea^2,3^. During the spring phytoplankton bloom, the CWC reef community is supported by the export of phytodetritus from the surface ocean^4–6^, but for significant parts of the year, the deep reefs are limited in particulate food sources^5,7^. Dissolved organic matter (DOM) could then act as an alternative C source (dissolved organic carbon, i.e. DOC). DOM is permanently present in the deep sea, but typically at low concentrations of <50 μΜ DOC^8^. However, mucus production by the CWCs and the metabolic activity of the dense reef epifauna, significantly increases the DOM concentration in the reef water^9–11^. The elevated, labile DOM measurably stimulates bacterial abundance and productivity^9,11,12^. Utilization of permanently present and enhanced DOM and bacteria within the reef community could retain energy on the reef that otherwise would be lost (Fig. 1a).

The faunal community on CWC reefs is characterized by a high abundance of active suspension feeders^13,14^. Three abundant species, especially on Norwegian CWC reefs, include the emergent high-microbial-abundance (HMA) sponge *Geodia barretti*, the emergent low-microbial-abundance (LMA) sponge *Mycale lingua*, and the bivalve *Acesta excavata*. *Mycale lingua* colonizes the live coral framework^15^, *G. barretti* lives on the dead reef framework or in sponge grounds neighbouring the reefs, and *A. excavata* forms clusters within grooves or beneath overhangs of the reef framework^15^. With their high water processing rates^16,17^, these active suspension feeders may readily access resources such as DOM and bacteria. Sponges pump water through their branched aquiferous system via beating flagella of the choanocytes, to retain bacterioplankton^16,18^, as well as DOM (reviewed by^19,20^). HMA sponges, formerly bacteriosponges^21,22^, have been considered particularly successful to access DOM with the aid of their associated microbial community^21,23,24^. Increasing evidence, however, suggests that LMA sponges, specifically encrusting species, likewise consume DOM^20,25^. Bivalves draw water into their enlarged gills (ctenidia^26^), where a moving, filtering mesh of feather-like latero-frontal cilia or cirri flicks particles from the water into a mucous string^27,28^. Few studies have addressed and confirmed the ability of freshwater, shallow-marine and hydrothermal-vent bivalves to utilize DOM and/or bacteria as substrates^29–32^.

The utilization of DOM and bacteria by active suspension feeders is a first step in resource retention within the reef community, however additional pathways may be active^33^. In the so-called sponge-loop^34^, sponges use coral-mucus-derived DOM, and in turn produce significant amounts of particulate detritus consisting of cellular debris^35^, which enters the detrital food chain^34,36^. Their high detritus production has been attributed to a high cell turnover and related cell-shedding^35,37,38^. Bivalves efficiently sort all ingested material before it enters their gut system, and release particulate material as (pseudo)feces^39–41^, which could likewise feed the detritivores in the reef community^42,43^. Utilization of waste material from the suspension feeders by reef detritivores (Fig. 1a) may act as a second step in the retention and recycling of resources within the reef community.

**Figure 2:**
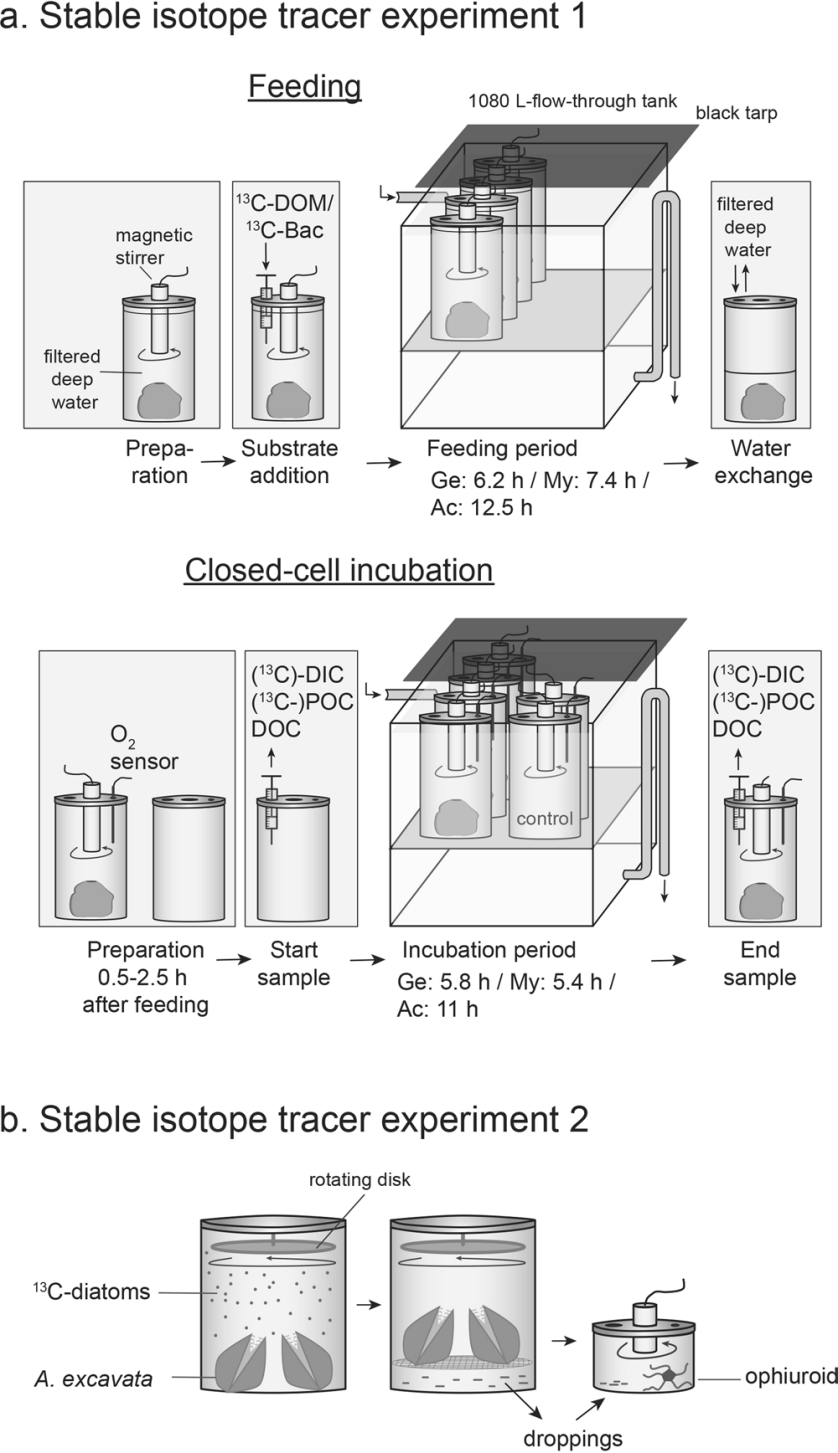
Set-up of stable isotope tracer experiments 1 and 2 (**a**,**b**). (**a**) Feeding of *G. barretti* (‘Ge’, in figure), *A. excavata* and *M. lingua*, (‘Ac’, ‘My’, not shown) with ^13^C-enriched dissolved organic matter (DOM) or ^13^C-enriched bacteria (‘Bac’). Subsequent closed-cell incubation, to measure O_2_ fluxes, total-C and tracer-C fluxes from concentration changes in O_2_ (sensor) and between start and end water samples for DIC (dissolved inorganic carbon), POC (particulate organic carbon), and DOC (dissolved organic carbon). (**b**) Feeding of *A. excavata* with ^13^C-enriched diatoms, collection of bivalve (pseudo-)fecal droppings and feeding of those to reef ophiuroids.

Here, we qualitatively evaluate the potential retention and subsequent recycling of DOM, bacteria and bivalve (pseudo-)feces within CWC reef communities (Fig. 1b), using a two-step experimental approach (Fig. 2). In stable isotope tracer experiment 1 (Fig. 2a), we studied the utilization of two substrates, ^13^C-enriched DOM (diatom-derived for logistical constraints, as explained below) and ^13^C-enriched bacteria by abundant CWC reef suspension feeders. Next to the HMA sponge *G. barretti*, the LMA sponge *M. lingua* and the bivalve *A. excavata* were chosen for this study, to (1) test the original hypothesis that HMA sponges, with their high abundance of microbes, are better-suited for DOM acquisition compared to other suspension feeders^21,23,24^, and (2) investigate whether bivalves, like sponges, can utilize bacteria as resource. The three taxa were fed in the laboratory with each substrate. Subsequently, the specimens were closed-cell incubated in filtered deep water without added DOM/bacteria, to measure (a) their utilization of substrate-derived tracer-C for respiration and detritus/(pseudo-)feces production (see tracer-C fluxes, i.e. ^13^C in Fig. 1b), and (b) their total respiration, and their total production of detritus/(pseudo-)feces and DOC waste (total-C fluxes, i.e. ^13^C + ^12^C). For (a), we traced ^13^C in the dissolved inorganic carbon (^13^C-DIC) and particulate organic carbon (^13^C-POC) released by the animals (tracer-C respiration, tracer POC release). For (b), we measured oxygen consumption, and total POC and DOC release. Finally, we traced ^13^C in the animal tissue (tracer-C incorporation). During the preparatory and experimental phase, we observed a particularly high production of (pseudo-)fecal droppings by *A. excavata*, which we considered as potential substrate for reef detritivores (Fig. 1a). This hypothesis was tested in a second stable isotope tracer experiment (Figs. 1b, 2b), in which we followed the isotope-tracer ^13^C through an ‘experimental food chain’, consisting of artificially ^13^C-enriched diatoms (*Skeletonema marinoi*), *A. excavata* fed with the diatoms, its (pseudo-)fecal droppings, and the tissue of reef ophiuroids fed with the bivalve droppings.

## Results

### Utilization of the substrates DOM and bacteria

**Figure 3:**
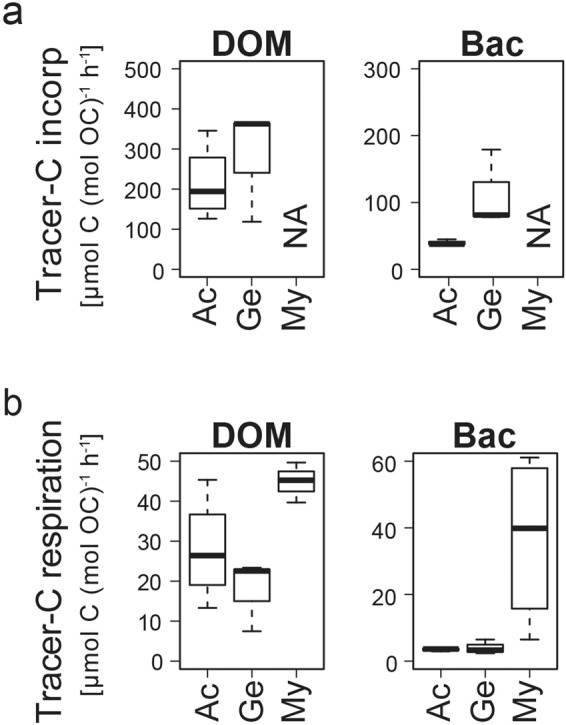
Utilization of DOM- and bacteria(‘Bac’)-tracer-C by *A. excavata* (Ac), *G. barretti* (Ge), and *M. lingua* (My). (**a**) Incorporation of tracer-C in the tissue of the animals, and (**b**) respiration of tracer-C.

All three investigated taxa utilized the substrates DOM and bacteria. *Geodia barretti* and *A. excavata* evidently incorporated DOM- and bacteria-tracer-C into their tissue (Fig. 3a). Tissue samples of *M. lingua* were unfortunately lost, so incorporation could not be evaluated for this species. However, all investigated taxa, *G. barretti*, *A. excavata* and *M. lingua*, respired DOM- and bacteria-tracer-C, as shown by their production of ^13^C-DIC during the closed-cell incubations (Fig. 3b).

*Geodia barretti* and *A. excavata* incorporated DOM-tracer-C at a similar rate (Fig. 3a), while *G. barretti* showed a higher average incorporation of bacteria-tracer-C than the bivalve, but this difference was not significant (Wilcoxon, p > 0.05, Supplementary Table S1). Results, however, indicate that *G. barretti* incorporated ~100% of the bacteria-tracer-C during the 6-h feeding period. Its bacteria-tracer-C incorporation rate is therefore likely an underestimate due to substrate depletion.

Both species incorporated DOM-tracer-C at a higher rate than bacteria-tracer-C (Wilcoxon, p < 0.05 only for *A. excavata*). It is, however, important to note that the DOM concentration in our experiment was seven times higher than the bacteria concentration, consistent with the concentration difference on the reefs in deep Norwegian fjords^24^.

*Mycale lingua* showed the highest tracer-C respiration rate for both substrates as compared to the other two taxa (Fig. 3b; Kruskall-Wallis, p < 0.05 only for bacteria). *Geodia barretti* and *A. excavata* showed lower, similar respiration rates of DOM-tracer-C and bacteria-tracer-C. DOM-tracer-C was respired at a higher rate than bacteria-tracer-C (Wilcoxon, p < 0.05 only for *A. excavata*).

### Production of particulate and dissolved organic matter

**Figure 4:**
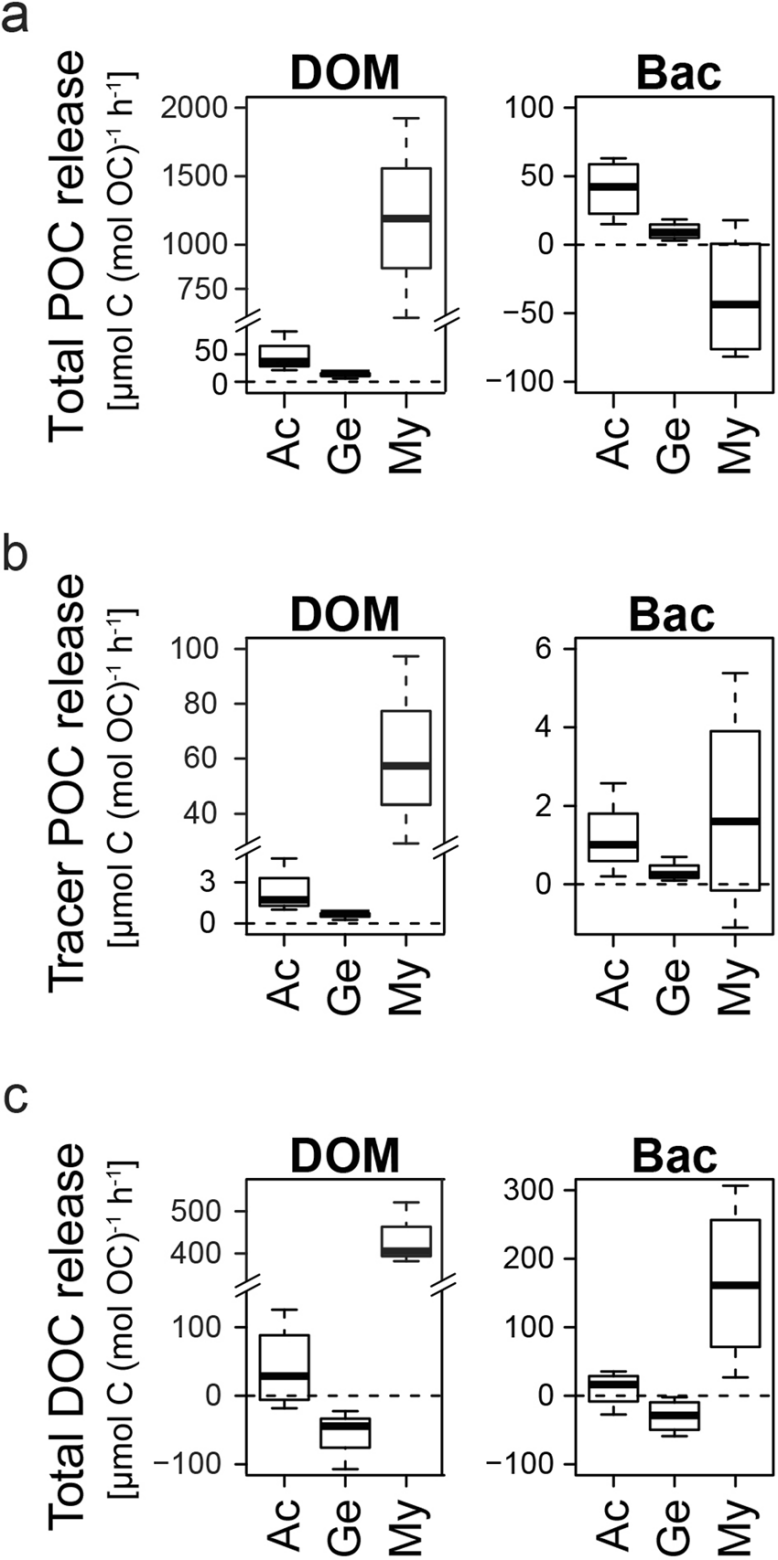
Production of organic waste by *A. excavata* (Ac), *G. barretti* (Ge), and *M. lingua* (My). Release of (**a**) total particulate organic carbon (POC), (**b**) tracer POC, and (**c**) total dissolved organic carbon (DOC). Release of tracer POC (**b**) indicates C derived from the DOM/bacteria-substrates, i.e. transformation of DOM/bacteria into sponge detritus/bivalve (pseudo)-feces. Please note the broken axes.

**Figure 5:**
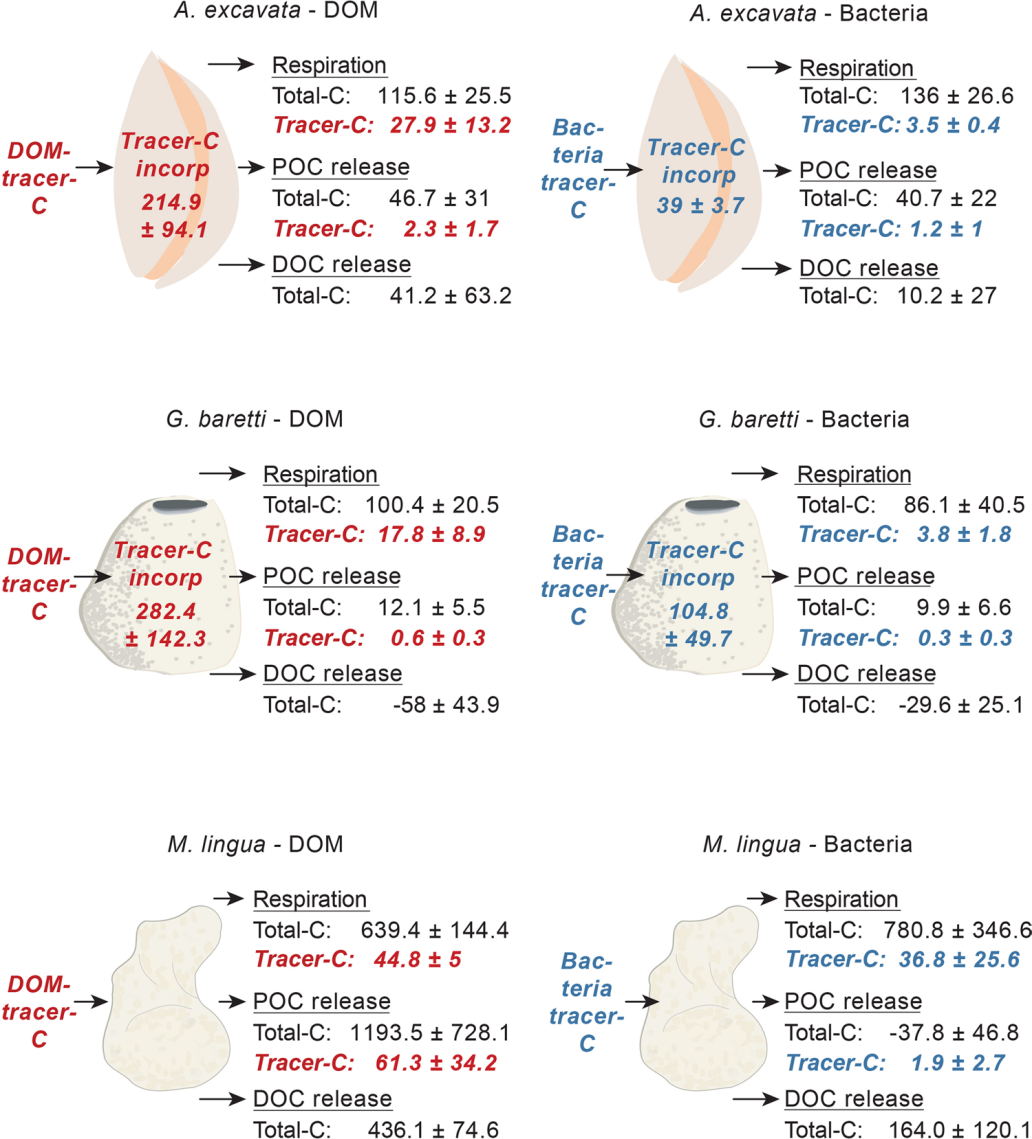
Budget of total-C and tracer-C fluxes [all in μmol C (mol OC)^−1^ h^−1^] of *A. excavata*, *G. barretti* and *M. lingua*, fed with either DOM (238 to 240 μM C) or bacteria (34 to 35 μM C). Total-C fluxes (in black) include the animals’ total-C respiration, total POC and total DOC release, and together add up to their total-C turnover. Negative values for DOC/POC release indicate a net uptake. Tracer-C fluxes (in red/blue) refer to the C which the animals utilized from the substrates DOM/bacteria, for tissue incorporation (‘Tracer-C incorp’) i.e. tissue growth, tracer-C respiration, and tracer POC release.

During the closed-cell incubation in filtered seawater following the substrate exposure period (i.e. without added DOM/bacteria), all experimental animals (besides previously bacteria-fed *M. lingua*) released particulate organic carbon (Figs. 4a, 5, total POC release). The released POC contained tracer-C from the previously consumed substrates DOM and bacteria (Figs. 4b, 5, tracer POC release), demonstrating that all investigated taxa had transformed parts of the substrate-C into detrital waste. *Acesta excavata* was characterized by a high release of total- and tracer POC (Fig. 5), which (largely) consisted of (pseudo-)fecal droppings (ca. 15 droppings bivalve^−1^ d^−1^). The droppings had a low buoyancy and visibly accumulated on the floor of the incubation chambers. *Geodia barretti*, however, had a low total- and tracer POC release (Figs. 4, 5), indicating a comparatively low production of particulate detritus (often termed ‘sponge detritus’ in sponge loop studies^34,36^). Total and tracer POC release by *M. lingua* was highly variable. DOM-fed *M. lingua* showed a high and variable total and tracer POC release. Bacteria-fed *M. lingua* in three out of four replicates took up total POC from the incubation water (Fig. 4a, background POC in the mix of filtered and unfiltered reef water). Tracer-POC, i.e. POC with C originating from the bacteria- or DOM-substrate, by contrary, was released (Fig. 4b).

During the incubations in filtered deep-sea water (DOC concentration of 73.5 μM), *A. excavata* and *M. lingua* showed a net release of DOC (Figs. 4c, 5), while *G. barretti* showed a detectable DOC uptake.

### Total-C and tracer-C budget

*Acesta excavata* and *G. barretti* incorporated the largest share (>88%) of the utilized bacteria-tracer-C and DOM-tracer-C in their tissue (Fig. 5, red/blue numbers), i.e. allocated it to tissue growth. A smaller fraction (4 to 12% of the tracer-C) was utilized for tracer-C respiration. Tracer POC release was the smallest sink of utilized tracer-C, < 3% in *A. excavata* and <0.3% in *G. barretti*. *Acesta excavata* released <0.11% of its tissue biomass per day as POC (Fig. 5, total POC release, black number), *G. barretti* < 0.03% d^−1^, and *M. lingua* < 2.87% d^−1^ (for tissue biomasses, see Supplementary Data S2).

### (Pseudo-)feces transfer to ophiuroids

**Figure 6:**
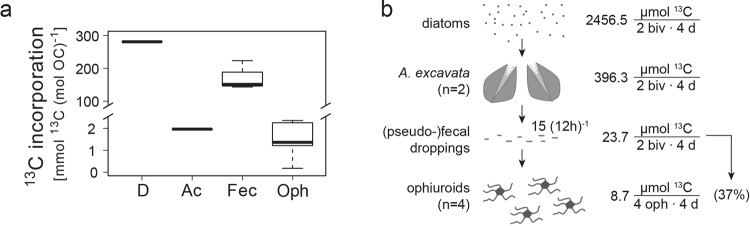
Recycling of bivalve (pseudo-)feces. (**a**) Tracer ^13^C, originating from artificially ^13^C-enriched diatoms (‘D’), incorporated in the tissue of the bivalve *A. excavata* (‘Ac’), in its (pseudo-)fecal droppings (‘Fec’), and in the tissue of reef ophiuroids fed with the droppings (‘Oph’). Please note the broken axis. (**b**) Four-day budget of ^13^C incorporated in the respective elements of this experimental food chain: The four ophiuroids (‘4 oph’) incorporated 37% of the (pseudo-)fecal ^13^C which the two bivalves (‘2 biv’) produced during the four experimental days (‘4 d’).

^13^C from the artificially-enriched diatom substrate (*Skeletonema marinoi*) could be traced in all parts of the experimental food chain (Fig. 6a), i.e. *A. excavata* tissue after consumption of the diatoms, the collected bivalve (pseudo-)fecal droppings, and the tissue of the CWC reef ophiuroids after exposure to (only) the *A. excavata* (pseudo-)fecal droppings. The four ophiuroids incorporated 37% of the (pseudo-)fecal ^13^C produced by two bivalves during the four experimental days (Fig. 6b).

## Discussion

Our study provides direct evidence that CWC reef sponges and bivalves are capable to retain and recycle DOM and bacteria, for tissue growth, metabolism, and detritus production. We will estimate the nutritional importance of the substrates for the suspension feeders. Further, we find that the bivalve *A. excavata*, more than the investigated sponges, acts as important detritus producer on CWC reefs, and demonstrate the utilization of its (pseudo-)fecal waste by detritivorous ophiuroids.

### DOM utilization

Sponges, in particular high-microbial-abundance (HMA) sponges, are considered as the dominant DOM consumers on both warm- and cold-water coral reefs^36,44^. Their close association with heterotrophic bacteria, which absorb dissolved substances more efficiently than invertebrates^45^, supposedly gives the HMA sponges an advantage in DOM utilization^21,23,46^. In the present study, the DOM incorporation rate of HMA sponge *G. barretti* is in the lower range of other cold-and warm-water sponges^36,47,48^. Surprisingly however, the CWC reef bivalve *A. excavata* and the low-microbial-abundance (LMA) sponge *M. lingua* show comparable or even higher rates of DOM-incorporation and/or metabolization (Figs. 3 and 5). The ability of marine invertebrates other than HMA sponges to utilize DOM as a substrate could indicate a direct DOM uptake in the animal cells, e.g. via specific membrane transporters for monosaccharides, amino acids or fatty acids, or via pinocytosis, i.e. the ingestion of liquid by membrane vesicle budding^19,49^. Several studies have provided evidence for DOM-C incorporation in both sponge and bacteria cells^36,47,50^. Nevertheless, CWC reef LMA sponges including *M. lingua* have a complex microbiome comparable to that of HMA sponges^51^, and the gills of *A. excavata* are inhabited by heterotrophic bacteria of the order *Oceanospirillales*^52^, which could likewise facilitate the indirect DOM uptake by the animal-microorganism holobiont.

In this experimental approach, we provided the animals with elevated concentrations of comparatively labile, artificial (diatom-derived) DOM. This DOM source may be taken-up at higher rates than natural DOM^50,53^. At the same time however, CWCs and other reef fauna actively release DOM^9,11^, and thereby increase the concentration of labile DOM on the reef^10^ above the typically low deep-sea DOC concentrations (<50 μM^8^). In a recirculating setting, Rix *et al*.^36^ demonstrated the utilization of CWC-derived DOM by the encrusting sponge *Hymedesmia coriacea*^36^. Unfortunately, a similar approach with CWC-derived DOM was logistically not feasible here, and the required quantities of ^13^C-enriched CWC mucus can realistically not be produced in a laboratory setting.

A recent study on Red Sea sponges has suggested a threshold concentration of 79 μM DOC, below which sponges cannot access DOC^54^. In our study however, *G. barretti* showed a net DOC uptake at a natural deep-fjord DOC concentration (73.5 μM DOC, Fig. 4). DOC uptake below the suggested threshold demonstrates that *G. barretti* is very efficient in exploiting this resource. *Mycale lingua* and *A. excavata*, by contrary, shift to a net DOC release at this low DOC concentration. Hence, these taxa likely profit only from enhanced DOM availability in specific reef microhabitats, e.g. in close vicinity to mucus-producing CWCs^9,36^, where specifically *M. lingua* frequently occurs^15^.

### Bacteria utilization

The CWC reef sponges incorporate and metabolize bacteria at a higher rate than the bivalve (Fig. 3). This is expected as sponges are known to retain bacteria with a near 100% efficiency^24,55,56^, while bivalves tend to target larger particles such as phytoplankton or phytodetritus (>4–7 μm^26^). *Acesta excavata* has an exceptionally high clearance rate for this larger particle size spectrum^57^. Specialization of suspension feeders on a certain particle size originates from the morphology and function of the filtration structure. The choanocyte-collar filter of sponges, a 0.06–2 μm-sized gasket- and mucus-sealed mesh^24,55,56^, efficiently retains bacteria-sized particles, while larger particles are phagocytosed by surface and canal-lining pinacocyte cells^19^. Bivalves, by contrary, do not have a ‘stiff’ mechanical filter, but a ‘paddling’ mesh of latero-frontal cilia^58^, which most efficiently traps larger particles, but allows the ‘by-catch’ of smaller bacteria, without being specialized on them.

### Nutritional importance of DOM and bacteria

The high fraction of DOM- and bacteria-tracer-C used for tissue growth (tracer-C incorporation), as compared to tracer-C respiration and tracer-POC release (Fig. 5), suggests that both substrates are of good nutritional quality for *G. barretti* and *A. excavata*. The present study, however, only provides a snapshot of the tracer-C utilization, as the partitioning of resources may change over time. Maier *et al*.^59^ measured tracer-C utilization of cold-water coral *Lophelia pertusa* at multiple time points, and demonstrated that, like *G. barretti* and *A. excavata*, the corals initially retained the majority of the acquired tracer-C in their tissue, from where it was utilized along with previously stored C.

All taxa utilize DOM-tracer-C at higher rates than bacteria-tracer-C (Fig. 5), which likely relates to the higher DOM concentration in the experiment. In the deep Norwegian fjords, the concentration of DOM-C is likewise higher than the concentration of bacteria-C^24^. Given the different substrate concentrations, it is difficult to identify substrate preferences for each of the taxa. Nevertheless, comparatively high bacteria-tracer-C utilization by *G. barretti* (100% incorporation) and *M. lingua* (high tracer-C respiration), in spite of the lower concentration, indicates that the sponges efficiently prey on bacteria. Correspondingly, a study by Leys *et al*.^24^ indicates that sponges are optimally-suited to exploit bacteria, but meet a large fraction of their C demand by naturally more abundant DOM. *Acesta excavata*, by contrary, incorporated bacteria-tracer-C at a lower rate compared to *G. barretti*, but DOM-tracer-C at a comparable rate. This suggests that the bivalve prefers other particulate substrates, such as phytodetritus, but can likewise meet its remaining nutritional demand by DOM consumption. The reliance of all species on DOM and bacteria remains to be tested under *in situ* conditions.

### Fuelling of the detritus food chain

Sponges are considered particularly important for the recycling capacity of warm- and cold-water coral reefs, due to their efficient (partial) transformation of DOM to sponge detritus, which fuels the detritivore food web^34–36^. The present results indicate that this so-called sponge-loop could be mediated by other suspension feeders which are abundant in CWC reef communities. All investigated sponges and bivalves partially transformed DOM (and bacteria) to organic particulates (>0.7 μm, tracer POC release, Fig. 4), and could hence mediate a detritivore recycling loop.

Nevertheless, compared to encrusting sponges, which release up to 40% of the assimilated DOM-derived tracer-C as sponge detritus^34,36^, the tracer POC release of *G. barretti*, *M. lingua* and *A. excavata* represents a small sink (<3%) of the utilized tracer-C (Fig. 5). The comparatively short incubation time may partly explain this low conversion. However, *G. barretti* also has a comparatively low total detritus production, and releases only 0.03% of its tissue-C d^−1^ as POC (Fig. 5). The low detritus production of *G. barretti*, accompanied by high investment of retained resources in tissue growth, matches the recently reported difference between emergent and encrusting sponges^60^. While emergent species like *G. barretti* can allocate a majority of assimilated C in three-dimensional tissue growth, their encrusting relatives are restricted to space-limited, two-dimensional growth, and may therefore invest the C over-supply in high cell turnover, cell shedding and detritus production^60^. The sponge loop on warm- and CWC reefs may thus be supported mostly by encrusting sponges, and hence be spatially confined to dead-coral-framework cavities^34,61^.

*M. lingua* shows a higher POC release (up to 2.9% tissue-C d^−1^), but the high variability indicates that this could partly be a measurement bias, as this very fragile species may be prone to tissue loss even when handled very carefully.

*Acesta excavata* could alternatively support the detritivore food chain, with a substantial release of POC as (pseudo-)fecal droppings. The bivalves occur in dense clusters of up to 23 individuals m^−2^ on the reefs (T. Kutti, unpublished data), and produce 2.6 to 6.3 μmol (pseudo-)fecal POC ind^−1^ h^−1^, depending on the substrate type and food concentration (Supplementary Data S2). The estimated (pseudo-)fecal POC release of 60 to 144 μmol C m^−2^ h^−1^ is comparable to the particulate mucus release by CWC *L. pertusa*^9^ (117 μmol C m^−2^ h^−1^). Due to their low buoyancy, the (pseudo-)fecal droppings sink fast (personal observation), and may accumulate below the *A. excavata* clusters on vertical and overhanging parts of CWC reefs. We show that the bivalve droppings are readily consumed by reef detritivorous ophiuroids. In this bivalve-driven recycling loop, the ophiuroids recycle 37% of the 4 d- (pseudo-)fecal waste of two bivalves for their own 4 d- tissue growth (Fig. 6). Their tissue incorporation (or assimilation) of 37% of the consumed detritus is in the range of other invertebrates detritivores^62–64^, and retains a significant amount of waste material in the live reef community. The quantitative importance of coral-derived DOM and bacteria to support this bivalve-driven recycling loop remains to be investigated, but we argue that C recycling appears to be an ubiquitous feature of main CWC reef components.

### Recycling pathways on CWC reefs

As ecosystem engineers, cold-water corals do not only alter their physical environment by creating a three-dimensional reef framework^62^, but also their biogeochemical environment. Their release of mucoid DOM elevates the DOM concentration in the reef water, and stimulates bacterial growth^9,10^. Our experimental study demonstrates that abundant reef suspension feeders, including sponges and bivalves, are able to retain concentrated, labile (diatom-derived) DOM and bacteria, and recycle it to biomass. Hereby, elevated DOM concentrations seem to profit all taxa, while the HMA sponge *G. barretti* can even access natural DOM at low, ambient deep fjord concentrations. Further, sponges preferably exploit bacteria, while *A. excavata* shows lower utilization of this resource. The bivalves likely prefer larger phytodetrital particles, indicating a niche separation between the suspension feeders based on particle size. We further show that particulate organic waste of the suspension feeders, specifically the substantial amount of (pseudo-)feces released by *A. excavata*, is consumed (recycled) by detritivorous reef ophiuroids. Efficient resource exploitation, and the close link between the suspension-feeding and the detritivore food web, may provide mechanisms for those deep-sea ecosystems to maintain high productivity, especially in the long periods of low phytodetritus availability between the settling of the spring plankton blooms^5,7^.

## Materials and Methods

### Collection and cultivation of reef organisms

Specimens of *G. barretti* and *A. excavata* were collected by remotely operated vehicle (ROV Aglantha, Institute of Marine Research, IMR) from 200 m depth on the Nakken reef, Hardanger fjord, Norway (59°49′N, 5°33′E), during the RV Hakon Mosby cruise 2016603 in February 2016. The animals were transported in ambient water in cooling boxes to the nearby aquarium facilities of the IMR Austevoll field station. They were maintained for two months in a 1080 L- tank with a flow-through of unfiltered deep fjord water. The deep fjord water was pumped from 165 m depth from an adjacent fjord arm, which is known to harbour both species (renewal rate: 1967 ± 58 L h^−1^; mean ± SD; temperature: 8.2 ± 0.2 °C, salinity: 35.1 ± 0.1‰, pH: 8.0 ± 0.1). For *G. barretti*, small whole individuals of 4–5 cm in diameter were used in the experiment. This is in contrast to the explant approach, which has commonly been used to study this species^65–67^. The advantage of non-explant, natural *G. barretti* specimens is their presence of oscula, an intact aquiferous system, and hence a natural pumping activity^24^ which was confirmed by fluorescein dye. Ophiuroids of the genus *Ophiacantha* (at least partly detritivorous^68^) were picked just prior to the experiment from pieces of coral framework (collection as *A. excavata*). *Mycale lingua* is a very fragile sponge, which cannot be kept in aquaria for long periods (personal observation). The experimental work on this species was therefore conducted onboard during RV GO SARS cruise 2016110 to Hola reef (Norway, 68°54′N, 14°23′E, 260 m depth) in July 2016. *Mycale lingua* was collected by ROV (Ægir 6000, NORMAR) and maintained onboard in a 1000 L- tank filled with *in situ* reef water, in a temperature-controlled room (7.5 °C). Water circulation was maintained with submersible pumps. Half the water was exchanged every 1–2 days. Only actively pumping sponges with open oscula were used in the experiment.

### Preparation of labelled substrates

Diatoms *(Skeletonema marinoi*, culture collection of the Royal Netherlands Institute for Sea Research, NIOZ) were cultured axenically on F/2-culture medium in 6 batches of twelve 1 L- flasks, with 2 mM NaHCO_3_ (99 atom% ^13^C), under a 12 h light −12 h dark cycle. Diatoms were harvested after 3 weeks^59^. Diatom cells were collected on a 0.45 μm-cellulose acetate filter, flushed into centrifuge tubes with artificial seawater, and concentrated by centrifugation. The diatom pellet was rinsed with ca. 1 L artificial seawater to remove residual medium, centrifuged and lyophilized. One part of the diatoms was used for DOM extraction for experiment 1, another was used in experiment 2. DOM was extracted in two batches from 2 g dry diatoms. Diatom cells were therefore lysed in ultrapure water. Cellular particulates were removed by centrifugation (4000 rpm). The supernatant DOM solution was filtered over 0.22 μm- sterile filters, and lyophilized. Mixed bacteria cultures were obtained in two batches from sediment (Oosterschelde mudflats, Netherlands), inoculated in 0.6 L unfiltered, aerated seawater with 0.8 M glucose, 0.8 M ammonium chloride, and yeast extract (17 °C, dark). 3 d-old culture medium was inoculated to new medium (8.3 M glucose, 1.875 M ammonium chloride, yeast extract), which after 3 d was transferred to the final M63 medium with ^13^C-glucose (U-^13^C_6_, 99 atom%) as C source. The bacteria were harvested after 3 d, by two-step centrifugation (2000 rpm to remove large cells/aggregates; 8500 rpm to obtain the small 1 μm-diameter cells). Individual bacteria pellets were rinsed with filtered seawater (FSW, 0.7 μm, 50 mL) to remove residual medium, and suspended in 1.5 mL FSW. All substrates were stored at −20 °C. Subsamples (diatoms: 1.5 mg, n = 3; DOM: 1 mg, n = 3; bacteria: 100 μL dried suspension, n = 2) were analyzed for C content and δ^13^C on an elemental analyser coupled to an isotope ratio mass spectrometer (EA-IRMS, Flash 1112, DELTA-V, THERMO, double resistors for measurement of highly ^13^C-enriched samples). L-glutamic acid (USGS40, USGS41), ^13^C-enriched glucose and bicarbonate were used as reference materials. The fractional ^13^C abundance of the substrates was: F^13^_diatoms_: 29.2%, F^13^_DOM_: 24.4 to 25.5%, F^13^_bacteria_: 94.7 to 96.5%). The EA-IRMS was thoroughly cleaned between analysis of highly ^13^C-enriched substrates and other samples (see below).

## Experiment 1: DOM and bacteria utilization

### Feeding

*Geodia barretti*, *A. excavata* and *M. lingua* specimens were fed separately with either DOM (238 to 240 μM C) or bacteria (34 to 35 μM C). Substrate concentrations were chosen high enough to ensure detectable isotope enrichment in metabolic products and tissue, but low enough to still resemble CWC reef concentrations^10^. The experimental DOM-C concentration was seven times higher than the bacteria-C concentration, a factor difference which is realistic for CWC reefs in the deep Norwegian fjords^24^. The animals were placed in 4.8 L-plexiglass incubation chambers (Fig. 2a, n = 4 per substrate, except for n = 3 for DOM-fed *G. barretti* and *M. lingua*), filled with fresh 0.35 μm-filtered deep fjord water (*A. excavata*, *G. barretti*, pumped from deep fjord) or 0.7 μm-filtered deep water (*M. lingua*, collected above reef with Niskin bottles). A magnetic stirrer in the chamber lid ensured mixing. The DOM and bacteria substrates were dissolved/suspended in 40 mL filtered deep water. Colloids were removed by forcing the bacteria solution through a 0.8 mm-syringe needle, and 0.22 μm-filtration of the DOM solution. The respective substrate was injected through a port in the lid. To maintain a stable temperature, the plexiglass incubation chambers were partially submerged in a tarp-covered (dark) 1080 L- flow-through tank (8.2 ± 0.2 °C); for the on-board experimental work on *M. lingua* in a 100 L-tank in a dark, 7.5 °C - temperature-controlled room. For each taxon, the feeding time was chosen as long as possible, to increase the chance of successful ^13^C-labelling. At the same time, feeding was stopped before the O_2_ saturation dropped below 80% to prevent adverse low-oxygen effects (*A. excavata* 12.5 ± 0.5 h, *G. barretti* 6.2 ± 0.2 h, *M. lingua*: 7.4 ± 1.3 h). The oxygen concentration was therefore monitored with a FireSting O_2_ logger (TeX4, Pyro Science). After feeding, the water in the feeding chambers was exchanged with filtered deep water to remove the residual ^13^C-labelled substrate. Between the feeding and the subsequent closed-cell incubation (see below), all chambers and sampling material were cleaned with 2% HCl.

### Closed-cell incubation

After the feeding (0.5 to 2.5 h), the animals were closed-cell incubated without food (Fig. 2a), to measure their total respiration, POC and DOC release, and the metabolization of the food substrates, as tracer-C respiration and tracer POC release. The animals were incubated in 1.3 L/4.8 L- plexiglass chambers, depending on their size. For each taxon, the incubation time was chosen as long as possible to detect the targeted C and O_2_ fluxes. At the same time, the incubations were stopped before the O_2_ saturation dropped below 80% to prevent adverse low-oxygen effects (*A. excavata*: 11 ± 1.6 h, *G. barretti*: 5.8 ± 1.2 h, *M. lingua:* 5.4 ± 1.2 h). *Geodia barretti* and *A. excavata* were incubated in fresh 0.35 μm-filtered deep fjord water, *M. lingua* in a mix of unfiltered and 0.7 μm-filtered deep water (filtration: glass fiber filters, i.e. GFF; unfiltered:filtered = 1:49; water collection see above). The incubation set-up was nearly identical to the feeding set-up, but incubation chambers were closed airtight and free from air bubbles. O_2_ consumption (respiration) rates of the incubated animals were derived from the O_2_ concentration decrease during the incubations, measured with a continuously logging FireSting probe fitted through the chamber lid. The release (production) of DIC (including ^13^C-DIC), POC (including ^13^C-POC) and DOC by the animals was measured as the increase in the respective concentration between a start and an end water sample. In the case of *G. barretti* and *A. excavata*, the start water samples were taken from an additionally-prepared chamber (no animal), and end water samples from each animal-chamber at the end of the incubation. In the case of *M. lingua*, the start samples were taken directly from each animal-chamber, and the removed water refilled with a known amount of 0.7 μm-filtered water, in which DIC, POC and DOC concentrations were also measured. DIC and DOC water samples were taken by glass syringe (2%-hydrochloric-acid [HCl]-cleaned). DIC samples were filled in 10 mL- headspace vials, fixed with 10 µL of a saturated mercury chloride solution and stored at 4 °C. DOC samples were filtered over pre-combusted (450 °C, 4 h) GFFs into acid-cleaned, pre-combusted amber vials (40 mL). DOC samples were acidified to pH 2 with concentrated HCl, and stored in the dark at 4 °C. For POC samples, a larger water volume (POC: 2 to 4.2 L, for *M. lingua*: 0.5 to 1 L) was filtered over pre-combusted, pre-weighed GFFs (per sample: one to three filters, i.e. subsamples), which were dried up to constant weight (40 °C), and stored dark at −20 °C. Two ‘no organism’- controls were run in parallel to each taxon-food-combination, to correct the animal O_2_ consumption, DIC, POC and DOC release rates.

### Animal sampling

After the incubations, i.e. after a total experimental time of 14 to 25 h, the animal volume was measured via water displacement in a graduated beaker. The tissue of sponges and bivalves (shells removed) was thoroughly rinsed with filtered seawater to remove non-ingested DOM/bacteria. The animal samples were lyophilized, and stored frozen (−20 °C). Additional samples of unfed *A. excavata* (n = 9) and *G. barretti* (n = 3) served to measure background isotope values. The *M. lingua* tissue samples were unfortunately lost, and data cannot be reported.

## Experiment 2: Transfer of (pseudo-)feces to ophiuroids

Two bivalves (*A. excavata*) were placed in one 7-L plexiglass chamber with 0.35 μm-filtered deep water, equipped with a rotating disk in the lid, and fed with ^13^C-enriched diatoms (300 μM C, Fig. 2b). After 12 h, the water was exchanged, and the bivalves were placed on a mesh. The bivalves produced distinct (pseudo-)fecal droppings which were collected after 12 h from the chamber floor with a pipette. The ophiuroids (n = 4) were placed in separate 1.2 L- plexiglass chambers with FSW. To each ophiuroid-chamber, three (pseudo-)fecal droppings were added, so that each ophiuroid was supplied with 7.2 ± 0.4 μmol C ophiuroid^−1^ d^−1^. After 22 h, the remaining (pseudo-)fecal droppings were removed and the water was exchanged. This cycle of bivalve-feeding, dropping-collection and ophiuroid-feeding was repeated four times (total C supply: 28.8 ± 1.6 μmol C ophiuroid^−1^ (4 d)^−1^). For the entire experiment, the respective incubation chambers were partially submerged in the 1080 L-flow-through tank to maintain a stable temperature (8.2 ± 0.2 °C). Ophiuroids, bivalves, and samples of bivalve (pseudo-)fecal droppings, collected on GFFs (n = 4), were lyophilized and stored frozen (−20 °C). Additional samples of unfed ophiuroids (n = 3), unfed bivalves (n = 9), and non-enriched droppings (n = 5) were analysed for background isotope values.

## Chemical analyses and calculations

### DIC, DOC and POC analysis

The DIC-δ^13^C was analysed by DIC-transformation to gaseous CO_2_ via addition of phosphoric acid, and CO_2_ injection on the EA-IRMS^59^ via an additional injection port downstream of the combustion tube. Total DIC concentration was measured on an Apollo SciTech AS-C3. DOC concentration was measured via high-temperature catalytic oxidation on a Shimadzu TOC-VCPN, with certified reference material (Hansell Laboratory). Total POC on the GFFs, and POC-δ^13^C, was analysed on the EA-IRMS described above (‘Preparation of labelled substrates’).

### Total-C and tracer-C fluxes

Total-C fluxes, measured in the closed-cell incubations, include the total-C respiration, estimated from the O_2_ consumption, assuming a respiratory quotient of O_2_:C = 1^69^, and the release (i.e. concentration change) of POC and DOC. Tracer-C fluxes, likewise measured in the closed-cell incubations, include tracer-C respiration and tracer POC release. Tracer-C fluxes were derived from the concentration change of ^13^C-DIC/^13^C-POC (calculated from the DIC/POC concentration change and the DIC/POC-δ^13^C), divided by the F^13^ of the respective substrate (see^59^ for details). Total-C and tracer-C fluxes were standardized to feeding/incubation time (hours) and tissue organic carbon content (OC, see next paragraph).

### Tissue organic carbon and tracer-C incorporation

*Acesta excavata*, *G. barretti*, and ophiuroid samples were homogenized by pestle and mortar. Subsamples (*A. excavata:* 2 mg, *G. barretti:* 5 mg, ophiuroids: 11 mg, n = 3 per sample) were analysed for tissue organic carbon content (OC), and δ^13^C on the EA-IRMS. Ophiuroid tissue was decalcified with HCl prior to the analysis^59^. The GFFs with (pseudo-)fecal droppings were analysed as a whole on the EA-IRMS. The amount of ^13^C in the animal tissue and bivalve droppings was calculated from the δ^13^C as described in detail in^59^, using Vienna Pee Dee Belemnite as standard with an isotope ratio of R = 0.0111802. Tracer-C incorporation was obtained from the amount of ^13^C divided by F^13^_substrates_, and standardized to the feeding time (hours) and tissue OC. The OC of *M. lingua* (required for the standardization of C fluxes) was estimated as 0.5 · AFDM^61,70^ (ash-free dry mass), their AFDM as AFDM = log(V) * 0.265 (T. Kutti, unpublished data), where V is the sponge volume. As an additional measure, we calculated the percentage of the provided tracer-C (i.e. bacteria- or DOM-tracer C added in the feeding incubations), which the animals incorporated.

### Data analysis

Data are reported as mean ± standard deviation. Data analysis was performed in R^71^. Non-parametric statistical testing was chosen to account for low replicate numbers. Detailed results of statistical tests are available as Supplementary Table S1. A Kruskal-Wallis-test with a Dunn post-hoc test (R package FSA^72^) was applied to compare tracer-C respiration and tracer POC release between *G. barretti*, *M. lingua* and *A. excavata*, fed with (1) DOM and (2) bacteria. A Wilcoxon rank sum test served to compare (a) rates of tracer-C incorporation between *G. barretti* and *A. excavata*, and (b) tracer-C incorporation, tracer-C respiration and tracer POC release of each taxon between the substrates DOM and bacteria.

## Supplementary information

Supplementary information.

Supplementary information 2.

## Data Availability

All relevant data are available as “Supplementary Data S2” and at 10.5281/zenodo. 3590262.

## References

[1] Freiwald, A. Reef-forming cold-water corals. In *Ocean Margin Systems* (eds. Wefer, G. *et al*.) 365–385 (Springer-Verlag Berlin Heidelberg, 2002).

[2] Van Oevelen, D. *et al*. The cold-water coral community as hotspot of carbon cycling on continental margins: a food-web analysis from Rockall Bank (northeast Atlantic). *Limnology and Oceanography* **54**, 1829–1844 (2009).

[3] Cathalot C (2015). Cold-water coral reefs and adjacent sponge grounds: hotspots of benthic respiration and organic carbon cycling in the deep sea. Frontiers in Marine Science.

[4] Thiem Ø, Ravagnan E, Fosså JH, Berntsen J (2006). Food supply mechanisms for cold-water corals along a continental shelf edge. Journal of Marine Systems.

[5] Duineveld GCA, Lavaleye MSS, Bergman MJN, De Stigter H, Mienis F (2007). Trophic structure of a cold-water coral mound community (Rockall Bank, NE Atlantic) in relation to the near-bottom particle supply and current regime. Bulletin of Marine Science.

[6] Soetaert, K., Mohn, C., Rengstorf, A., Grehan, A. & Van Oevelen, D. Ecosystem engineering creates a direct nutritional link between 600-m deep cold-water coral mounds and surface productivity. *Scientific Reports* **6**, 35057 (2016). 10.1038/srep35057 PMC5057138 27725742

[7] Duineveld GCA, Lavaleye MSS, Berghuis EM (2004). Particle flux and food supply to a seamount cold-water coral community (Galicia Bank, NW Spain). Marine Ecology Progress Series.

[8] Hansell DA, Carlson CA (1998). Deep-ocean gradients in the concentration of dissolved organic carbon. Nature.

[9] Wild C (2008). Organic matter release by cold water corals and its implication for fauna-microbe interaction. Marine Ecology Progress Series.

[10] Wild C (2009). Microbial degradation of cold-water coral-derived organic matter: potential implication for organic C cycling in the water column above Tisler Reef. Aquatic Biology.

[11] Van Bleijswijk, J. D. L. *et al*. Microbial assemblages on a cold-water coral mound at the SE Rockall Bank (NE Atlantic): interactions with hydrography and topography. *Biogeosciences* **12**, 4483–4496 (2015).

[12] Jensen S, Bourne DG, Hovland M, Colin Murrell J (2012). High diversity of microplankton surrounds deep-water coral reef in the Norwegian Sea. FEMS Microbiol Ecol.

[13] Roberts, J. M. *et al*. Monitoring environmental variability around cold-water coral reefs: the use of a benthic photolander and the potential of seafloor observatories. in *Cold-Water Corals and Ecosystems* (eds. Freiwald, A. & Roberts, J. M.) 483–502, 10.1007/3-540-27673-4_24 (Springer Berlin Heidelberg, 2005).

[14] Henry L-A, Davies AJ, Roberts JM (2010). Beta diversity of cold-water coral reef communities off western Scotland. Coral Reefs.

[15] Purser A (2013). Local variation in the distribution of benthic megafauna species associated with cold-water coral reefs on the Norwegian margin. Continental Shelf Research.

[16] Riisgård HU, Larsen PS (1995). Filter-feeding in marine macro-invertebrates: pump characteristics, modelling and energy cost. Biological Reviews.

[17] Gili J-M, Coma R (1998). Benthic suspension feeders: their paramount role in littoral marine food webs. Trends in Ecology & Evolution.

[18] Larsen PS, Riisgård HU (1994). The sponge pump. Journal of Theoretical Biology.

[19] Maldonado, M., Ribes, M. & Van Duyl, F. C. Nutrient fluxes through sponges: Biology, budgets, and ecological implications. in *Advances in marine biology* vol. 62 113–182 (Academic Press, 2012). 10.1016/B978-0-12-394283-8.00003-5 22664122

[20] De Goeij, J. M., Lesser, M. P. & Pawlik, J. R. Nutrient fluxes and ecological functions of coral reef sponges. In *a changing ocean in Climate change, ocean acidification and sponges: impacts across multiple levels of organization* (eds. Carballo, J. L. & Bell, J. J.) 373–410, 10.1007/978-3-319-59008-0_8 (Springer, 2017).

[21] Reiswig HM (1981). Partial carbon and energy budgets of the bacteriosponge *Verohgia fistularis* (Porifera: Demospongiae) in Barbados. Marine Ecology.

[22] Hentschel U, Usher KM, Taylor MW (2006). Marine sponges as microbial fermenters. FEMS Microbiol Ecol.

[23] Yahel G, Sharp JH, Marie D, Häse C, Genin A (2003). *In situ* feeding and element removal in the symbiont-bearing sponge *Theonella swinhoei*: bulk DOC is the major source for carbon. Limnology and Oceanography.

[24] Leys SP, Kahn AS, Fang JKH, Kutti T, Bannister RJ (2018). Phagocytosis of microbial symbionts balances the carbon and nitrogen budget for the deep‐water boreal sponge *Geodia barretti*. Limnology and Oceanography.

[25] Pawlik, J. R. & McMurray, S. E. The emerging ecological and biogeochemical importance of sponges on coral reefs. *Ann Rev Mar Sci*, 10.1146/annurev-marine-010419-010807 (2019). 10.1146/annurev-marine-010419-010807 31226028

[26] Ward JE, Shumway SE (2004). Separating the grain from the chaff: particle selection in suspension- and deposit-feeding bivalves. Journal of Experimental Marine Biology and Ecology.

[27] Riisgård HU, Larsen PS, Nielsen NF (1996). Particle capture in the mussel *Mytilus edulis*: the role of latero-frontal cirri. Mar. Biol..

[28] Ward, J. E., Sanford, L. P., Newell, R. I. E. & MacDonald, B. A. A new explanation of particle capture in suspension-feeding bivalve molluscs. *Limnology and Oceanography* **43**, 741–752 (1998).

[29] Sorokin YI, Wyshkwarzev DI (1973). Feeding on dissolved organic matter by some marine animals. Aquaculture.

[30] Amouroux JM (1986). Comparative study of the carbon cycle in *Venus verrucosa* fed on bacteria and phytoplankton. Mar. Biol..

[31] Roditi HA, Fisher NS, Sanudo-Wilhelmy SA (2000). Uptake of dissolved organic carbon and trace elements by zebra mussels. Nature.

[32] Fiala-Médioni A, Alayse AM, Cahet G (1986). Evidence of *in situ* uptake and incorporation of bicarbonate and amino acids by a hydrothermal vent mussel. Journal of Experimental Marine Biology and Ecology.

[33] Crossland CJ, Hatcher BG, Smith SV (1991). Role of coral reefs in global ocean production. Coral Reefs.

[34] De Goeij, J. M. *et al*. Surviving in a marine desert: the sponge loop retains resources within coral reefs. *Science* **342**, 108–110 (2013). 10.1126/science.1241981 24092742

[35] De Goeij, J. M. *et al*. Cell kinetics of the marine sponge *Halisarca caerulea* reveal rapid cell turnover and shedding. *The* *Journal of Experimental Biology* **212**, 3892–3900 (2009). 10.1242/jeb.034561 19915132

[36] Rix L (2016). Coral mucus fuels the sponge loop in warm- and cold-water coral reef ecosystems. Scientific Reports.

[37] Alexander BE (2014). Cell turnover and detritus production in marine sponges from tropical and temperate benthic ecosystems. PLoS ONE.

[38] Alexander BE (2015). Cell kinetics during regeneration in the sponge *Halisarca caerulea*: how local is the response to tissue damage?. PeerJ.

[39] Tsuchiya M (1980). Biodeposit production by the mussel *Mytilus edulis* L. on rocky shores. Journal of Experimental Marine Biology and Ecology.

[40] Beninger PG, Ward JE, MacDonald BA, Thompson RJ (1992). Gill function and particle transport in *Placopecten magellanicus* (Mollusca: Bivalvia) as revealed using video endoscopy. Marine Biology.

[41] Ward EJ, MacDonald BA (1996). Pre-ingestive feeding behaviors of two sub-tropical bivalves (*Pinctada imbricata* and *Arca zebra*): responses to an acute increase in suspended sediment concentration. Bulletin of Marine Science.

[42] Wotton RS, Malmqvist B (2001). Feces in Aquatic Ecosystems. BioScience.

[43] Rothans TC, Miller AC (1991). A link between biologically imported particulate organic nutrients and the detritus food web in reef communities. Mar. Biol..

[44] De Goeij JM (2008). Major bulk dissolved organic carbon (DOC) removal by encrusting coral reef cavity sponges. Marine Ecology Progress Series.

[45] Siebers D (1982). Bacterial-invertebrate interactions in uptake of dissolved organic matter. Integr Comp Biol.

[46] Ribes M (2012). Functional convergence of microbes associated with temperate marine sponges. Environmental Microbiology.

[47] De Goeij, J. M., Moodley, L., Houtekamer, M., Carballeira, N. M. & Van Duyl, F. C. Tracing ^13^C-enriched dissolved and particulate organic carbon in the bacteria-containing coral reef sponge *Halisarca caerulea*: evidence for DOM-feeding. *Limnology and Oceanography* **53**, 1376–1386 (2008).

[48] Rix L (2018). Reef sponges facilitate the transfer of coral-derived organic matter to their associated fauna via the sponge loop. Marine Ecology Progress Series.

[49] Wright SH, Manahan DT (1989). Integumental nutrient uptake by aquatic organisms. Annual Review of Physiology.

[50] Rix L (2017). Differential recycling of coral and algal dissolved organic matter via the sponge loop. Functional Ecology.

[51] Schöttner S (2013). Relationships between host phylogeny, host type and bacterial community diversity in cold-water coral reef sponges. PLoS ONE.

[52] Jensen S, Duperron S, Birkeland N-K, Hovland M (2010). Intracellular *Oceanospirillales* bacteria inhabit gills of *Acesta* bivalves. FEMS Microbiol Ecol.

[53] Jørgensen CB (1976). August Pütter, August Krogh, and modern ideas on the use of dissolved organic matter in aquatic environments. Biological Reviews.

[54] Wooster MK, McMurray SE, Pawlik JR, Morán XAG, Berumen ML (2019). Feeding and respiration by giant barrel sponges across a gradient of food abundance in the Red Sea. Limnology and Oceanography.

[55] Reiswig HM (1974). Water transport, respiration and energetics of three tropical marine sponges. Journal of Experimental Marine Biology and Ecology.

[56] Pile, A. J., Patterson, M. R. & Witman, J. D. *In situ* grazing on plankton <10 µm by the boreal sponge *Mycale lingua*. *Marine Ecology Progress Series* **141**, 95–102 (1996).

[57] Järnegren J, Altin D (2006). Filtration and respiration of the deep living bivalve *Acesta excavata* (J.C. Fabricius, 1779) (Bivalvia; Limidae). Journal of Experimental Marine Biology and Ecology.

[58] Wright RT, Coffin RB, Ersing CP, Pearson D (1982). Field and laboratory measurements of bivalve filtration of natural marine bacterioplankton. Limnology and Oceanography.

[59] Maier SR (2019). Survival under conditions of variable food availability: resource utilization and storage in the cold-water coral *Lophelia pertusa*. Limnology and Oceanography.

[60] McMurray SE, Stubler AD, Erwin PM, Finelli CM, Pawlik JR (2018). A test of the sponge-loop hypothesis for emergent Caribbean reef sponges. Marine Ecology Progress Series.

[61] Richter C, Wunsch M, Rasheed M, Kötter I, Badran MI (2001). Endoscopic exploration of Red Sea coral reefs reveals dense populations of cavity-dwelling sponges. Nature.

[62] Ginger ML (2001). Organic matter assimilation and selective feeding by holothurians in the deep sea: some observations and comments. Progress in Oceanography.

[63] Gergs R, Rothhaupt K-O (2008). Feeding rates, assimilation efficiencies and growth of two amphipod species on biodeposited material from zebra mussels. Freshwater Biology.

[64] Welch HE (1968). Relationships between assimiliation efficiencies and growth efficiencies for aquatic consumers. Ecology.

[65] Hoffmann F, Rapp HT, Zöller T, Reitner J (2003). Growth and regeneration in cultivated fragments of the boreal deep water sponge *Geodia barretti* Bowerbank, 1858 (Geodiidae, Tetractinellida, Demospongiae). Journal of Biotechnology.

[66] Hoffmann F (2009). Complex nitrogen cycling in the sponge *Geodia barretti*. Environmental Microbiology.

[67] Fang JKH (2018). Impact of particulate sediment, bentonite and barite (oil-drilling waste) on net fluxes of oxygen and nitrogen in Arctic-boreal sponges. Environmental Pollution.

[68] Allen Brooks R, Nizinski MS, Ross SW, Sulak KJ (2007). Frequency of sublethal injury in a deepwater ophiuroid, *Ophiacantha bidentata*, an important component of western Atlantic *Lophelia* reef communities. Mar Biol.

[69] Glud RN, Eyre BD, Patten N (2008). Biogeochemical responses to mass coral spawning at the Great Barrier Reef: effects on respiration and primary production. Limnology and Oceanography.

[70] Piepenburg D, Schmid MK (1997). A photographic survey of the epibenthic megafauna of the Arctic Laptev Sea shelf: distribution, abundance, and estimates of biomass and organic carbon demand. Marine Ecology Progress Series.

[71] R Core Team. *R: A language and environment for statistical computing. R Foundation for Statistical Computing, Vienna, Austria. URL* https://www.R-project.org/. (2017).

[72] Ogle, D. H., Wheeler, P. & Dinno, A. *FSA: Fisheries Stock Analysis. R package version 0.8.22*, https://github.com/droglenc/FSA (2018).

