# Supplementary material for: Recycling pathways in cold-water coral reefs: Use of dissolved organic matter and bacteria by key suspension feeding taxa
Source: Sci Rep. 2020 Jun 18;10:9942. doi: 10.1038/s41598-020-66463-2 (PMC7303112; doi:10.1038/s41598-020-66463-2)
Supplement: Supplementary file 1 — Supplementary information. [file 41598_2020_66463_MOESM1_ESM.docx]

Title of article:

Recycling pathways in cold-water coral reefs: Use of dissolved organic matter and bacteria by key suspension feeding taxa

Maier, Sandra R.^1^*; Kutti, Tina^2^; Bannister, Raymond J.^2^; Fang, James K.H.^3^; van Breugel, Peter^1^; van Rijswijk, Pieter^1^; van Oevelen, Dick^1^*

1: Department of Estuarine and Delta Systems, Royal Netherlands Institute for Sea Research (NIOZ-Yerseke) and Utrecht University, Yerseke, The Netherlands

2: IMR Institute of Marine Research, Nordnesgaten 50, 5005 Bergen, Norway

3: Department of Applied Biology and Chemical Technology, The Hong Kong Polytechnic University, Hung Hom, Kowloon, Hong Kong

Supplementary Table S1: Results of two-sided Wilcoxon tests, and Kruskal-Wallis tests with Dunn post-hoc tests. Ac: *A. excavata*, Ge: *G. barretti*, My: *M. lingua*. AcDOM: *A. excavata* fed with DOM, AcBac: *A. excavata* fed with bacteria, and respectively for GeDOM, GeBac, MyDOM, MyBac. df: degrees of freedom, p: p-value for Kruskal-Wallis or Wilcoxon test, *: significant difference p < 0.05, P unadj/adj: unadjusted and adjusted p-value for Dunn tests, only noted when significant as P_adj_ > 0.05.

| **Parameter** | **Unit** | **compared** | **test (non-par.)** | **W/chi2** | **df** | **p** | **sign diff between** | **Z** | | **P unadj** | | **P adj** | |
| --- | --- | --- | --- | --- | --- | --- | --- | --- | --- | --- | --- | --- | --- |
| Tracer-C incor-poration | μmol tracer C (mol OC)^-1^ h^-1^ | AcDOM, GeDOM | wilcoxon, 2 sided | 4.00 |  | 0.63 | NA | | | | | | |
|  |  | AcBac, GeBac | wilcoxon, 2 sided | 0.00 |  | 0.03* | NA | | | | | | |
|  | μmol tracer C (mol OC)^-1^ h^-1^ | AcDOM, AcBac | wilcoxon, 2 sided | 16.00 |  | 0.03* | NA | | | | | | |
|  |  | GeDOM, GeBac | wilcoxon, 2 sided | 11.00 |  | 0.11 | NA | | | | | | |
| Tracer-C respiration | μmol tracer C (mol OC)^-1^ h^-1^ | AcDOM, GeDOM, MyDOM | kruskal.wallis | 5.25 | 2 | 0.07 | NA | | | | | | |
|  |  | AcBac, GeBac, MyBac | kruskal.wallis | 6.58 | 2 | 0.04* | dunn not significant | | | | | | |
|  |  | AcDOM, AcBac | wilcoxon, 2 sided | 16.00 |  | 0.03* | NA | | | | | | |
|  |  | GeDOM, GeBac | wilcoxon, 2 sided | 2.00 |  | 0.06 | NA | | | | | | |
|  |  | MyDOM, MyBac | wilcoxon, 2 sided | 6.00 |  | 1 | NA | | | | | | |
| Tracer POC Release | μmol tracer C (mol OC)^-1^ h^-1^ | AcDOM, GeDOM, MyDOM | kruskal.wallis | 8.02 | 2 | 0.02* | GeDOM, MyDOM | | -2.83 | | 0.00 | | 0.01 |
|  |  | AcBac, GeBac, MyBac | kruskal.wallis | 2.35 | 2 | 0.31 | NA | | | | | | |
|  |  | AcDOM, AcBac | wilcoxon, 2 sided | 12.00 |  | 0.34 | NA | | | | | | |
|  |  | GeDOM, GeBac | wilcoxon, 2 sided | 11.00 |  | 0.11 | NA | | | | | | |
|  |  | MyDOM, MyBac | wilcoxon, 2 sided | 12.00 |  | 0.06 | NA | | | | | | |
